# Draft Genome Sequences of the Type Strains of Three *Clavibacter* Subspecies and Atypical Peach-Colored Strains Isolated from Tomato

**DOI:** 10.1128/MRA.01357-18

**Published:** 2018-11-08

**Authors:** Ebrahim Osdaghi, Perrine Portier, Martial Briand, Géraldine Taghouti, Marie-Agnes Jacques

**Affiliations:** a Department of Plant Protection, College of Agriculture, Shiraz University, Shiraz, Iran; b INRA, Agrocampus-Ouest, SFR 4207 QUASAV, Université d’Angers, Beaucouzé, France; c CIRM-CFBP, French Collection of Plant-Associated Bacteria, INRA, Beaucouzé, France; Georgia Institute of Technology

## Abstract

Here, we present the draft genome sequences of 10 Clavibacter sp. strains, including the type strains of different subspecies of Clavibacter michiganensis and a potentially novel species within the genus. Genome lengths of the strains varied between 2,982,864 and 3,288,331 bp, with G+C contents of 72.23 to 73.50%.

## Abstract (precis)

Here, we present the draft genome sequences of 10 Clavibacter sp. strains, including the type strains of different subspecies of Clavibacter michiganensis and a potentially novel species within the genus.

## ANNOUNCEMENT

The genus Clavibacter (Microbacteriaceae) comprises Gram-positive actinobacteria, most of which are pathogenic on agriculturally important crops (1). Traditionally, the genus Clavibacter has been considered to include only one species, Clavibacter michiganensis, comprising five plant-pathogenic subspecies, i.e., C. michiganensis subsp. insidiosus, C. michiganensis subsp. michiganensis, C. michiganensis subsp. nebraskensis, C. michiganensis subsp. sepedonicus, and C. michiganensis subsp. tessellarius (2). Recently, plant-associated but nonpathogenic members of C. michiganensis were assigned to new subspecies, including C. michiganensis subsp. californiensis and C. michiganensis subsp. chilensis (3). Additionally, C. michiganensis subsp. phaseoli and C. michiganensis subsp. capsici were identified as the causal agents of bacterial bean leaf yellowing and bacterial canker of pepper, respectively (4, 5). On the other hand, peach color-pigmented nonpathogenic Clavibacter sp. strains were isolated from tomato phyllosphere and remain to be taxonomically evaluated (6, 7).

Recently, a reclassification of C. michiganensis members into two new species and three new combinations was proposed (8). However, due to the lack of genomic information from every newly identified subspecies, further investigations are warranted to clarify the taxonomy of Clavibacter spp. Here, we present the draft genome sequences of 10 Clavibacter species strains (Table 1), including the type strains of C. michiganensis subsp. californiensis (CFBP 8216), C. michiganensis subsp. chilensis (CFBP 8217), and C. michiganensis subsp. phaseoli (CFBP 8627), as well as the atypical peach-colored strains (CFBP 8615 and CFBP 8616) isolated from tomato in Iran (6, 7). The strains CFBP 8615 and CFBP 8616 resulted from the screening of microbial communities associated with tomato plants at Shiraz University in Iran (7, 9) and were isolated from asymptomatic tomato leaves on yeast extract-peptone-glucose agar (YPGA) medium as previously described (6), whereas the remaining eight strains were provided by CIRM-CFBP in France (2).

**TABLE 1:** Source, place, and date of isolation, as well as genome information, for each *Clavibacter* species strain used in this study

Nomenclature	CIRM-CFBP^*a*^ code	Host of isolation	Yr of isolation	Country of isolation	Genome information							Accession no.			Reference or source
					G+C content (%)	Genome length (bp)	No. of contigs	Sequencing coverage (×)	No. of protein- coding genes	No. of RNA genes	No. of pseudogenes	DDBJ/ENA/ GenBank	Sequence Read Archive		
													Run	Expt	
Clavibacter michiganensis subsp. insidiosus	CFBP 1195	Medicago sativa	1964	United Kingdom	72.84	3,203,470	805	450	3,333	52	199	QWDZ01000000	SRR7977544	SRX4810326	16
Clavibacter michiganensis subsp. insidiosus	CFBP 6488	Medicago sativa	1998	Czech Republic	72.23	3,225,729	1,892	435	3,890	52	329	QWEA01000000	SRR7977605	SRX4810381	2
Clavibacter michiganensis	CFBP 7491	Solanum lycopersicum	ND^*b*^	ND	73.02	3,288,331	921	475	3,560	51	122	QWEB01000000	SRR7977581	SRX4810357	This study
Clavibacter michiganensis	CFBP 7493	Solanum lycopersicum	ND	ND	72.91	3,275,884	782	570	3,487	51	91	QWEC01000000	SRR7977687	SRX4810463	This study
Clavibacter michiganensis subsp. nebraskensis	CFBP 7577	Zea mays	ND	ND	72.75	2,982,864	1,273	510	3,291	53	373	QWED01000000	SRR7977688	SRX4810464	2
Clavibacter michiganensis subsp. californiensis	CFBP 8216^T^	Solanum lycopersicum	2000	United States (Hawaii)	72.71	3,193,415	811	515	3,367	51	105	QWEE01000000	SRR7983517	SRX4814786	3
Clavibacter michiganensis subsp. chilensis	CFBP 8217^T^	Solanum lycopersicum	2007	Netherlands	73.50	3,044,807	1,002	450	3,356	54	114	QWGS01000000	SRR7983516	SRX4814785	3
Clavibacter spp.	CFBP 8615	Solanum lycopersicum	2015	Iran	73.23	3,129,097	620	580	3,236	50	78	QWGT01000000	SRR7983538	SRX4814807	6
	CFBP 8616	Solanum lycopersicum	2015	Iran	73.15	3,094,686	961	555	3,342	50	151	QWGU01000000	SRR7983541	SRX4814809	6
Clavibacter michiganensis subsp. phaseoli	CFBP 8627^T^	Phaseolus vulgaris	2009	Spain	73.46	3,052,098	1,009	460	3,289	53	205	QWGV01000000	SRR7983540	SRX4814808	4

a CIRM-CFBP, International Center for Microbial Resources—French Collection of Plant-Associated Bacteria, IRHS UMR 1345 INRA-ACO-UA, Beaucouzé, France (https://www6.inra.fr/cirm_eng/CFBP-Plant-Associated-Bacteria).

b ND, not determined.

The Clavibacter sp. strains (Table 1) were grown on YPGA medium as previously described (2), and DNAs were extracted using the Wizard genomic DNA purification kit (Promega, Madison WI). The DNAs were sequenced using the Illumina HiSeq X platform at BGI Tech Solutions (Hong Kong), and the shotgun sequencing yielded 150-bp paired-end reads. A combination of Velvet (v 1.2.10), SOAPdenovo (v 2.04), and SOAPGapCloser (v 1.12) platforms (10, 11) was used for genome assembly. The genomes were assembled using the same strategy and the default settings of the platforms. In brief, different values of kmers were tested to obtain a preassembly with SOAPdenovo. Then, the resulting preassemblies were treated as long reads and introduced in Velvet to obtain final assemblies. Libraries were prepared using 170 to 800 bp. For each strain, genome length (bp) and G+C content (%) are summarized in Table 1. Additionally, genome annotation was performed using the GeneMarkS+ (v 4.6) suite implemented in the NCBI Prokaryotic Genome Annotation Pipeline with default settings (12). Total numbers of protein-coding genes, RNA genes, and pseudogenes were determined for all the genomes, as shown in Table 1.

Average nucleotide identity (ANI) analysis using the JSpeciesWS Web server (13) showed that the atypical peach-colored strains CFBP 8615 and CFBP 8616 have only 89.00 to 93.00% sequence identity with the type strains of previously identified and/or newly introduced Clavibacter species/subspecies. These ANI values are far below the accepted threshold (95 to 96%) for the definition of prokaryotic species (14), suggesting that the strains CFBP 8615 and CFBP 8616 could be defined as a new species. A comprehensive multiphasic taxonomic study using the genome sequences provided in this study to reevaluate the taxonomy of Clavibacter spp. and clarify the position of the strains CFBP 8615 and CFBP 8616 within the genus is ongoing.

### Data availability.

These whole-genome shotgun projects have been deposited at DDBJ/EMBL/GenBank under the accession numbers shown in Table 1. For all sequences, the first versions of the accession numbers are described in this paper. The raw reads of the sequences of all strains were submitted to the Sequence Read Archive (SRA) database (15), and the corresponding accession numbers are shown in Table 1. All of the strains listed in Table 1 are available at CIRM-CFBP, the French Collection of Plant-Associated Bacteria (http://www6.inra.fr/cirm_eng/CFBP-Plant-Associated-Bacteria).
